# SeqClone: sequential Monte Carlo based inference of tumor subclones

**DOI:** 10.1186/s12859-018-2562-y

**Published:** 2019-01-05

**Authors:** Oyetunji E. Ogundijo, Xiaodong Wang

**Affiliations:** Department of Electrical Engineering, Columbia University, New York, NY 10027 USA

**Keywords:** Tumor heterogeneity, Bayesian model, Sequential Monte Carlo, Indian buffet process

## Abstract

**Background:**

Tumor samples are heterogeneous. They consist of varying cell populations or subclones and each subclone is characterized with a distinct single nucleotide variant (SNV) profile. This explains the source of genetic heterogeneity observed in tumor sequencing data. To make precise prognosis and design effective therapy for cancer, ascertaining the subclonal composition of a tumor is of great importance.

**Results:**

In this paper, we propose a state-space formulation of the feature allocation model. This model is interpreted as the blind deconvolution of the expected variant allele fractions (VAFs). VAFs are deconvolved into a binary matrix of genotypes and a matrix of genotype proportions in the samples. Specifically, we consider a sequential construction of the genotype matrix which we model by Indian buffet process (IBP). We describe an efficient sequential Monte Carlo (SMC) algorithm, SeqClone, that jointly estimates the genotypes of subclones and their proportions in the samples. When compared to other methods for resolving tumor heterogeneity, SeqClone provides comparable and sometimes, better estimates of model parameters. By design, SeqClone conveniently handles any number of probed SNVs in the samples. In particular, we can analyze VAFs from newly probed SNVs to improve existing estimates, an attribute not present in existing solutions.

**Conclusions:**

We show that the SMC algorithm for deconvolving VAFs from tumor sequencing data is a robust and promising alternative for explaining the observed genetic heterogeneity in tumor samples.

**Electronic supplementary material:**

The online version of this article (10.1186/s12859-018-2562-y) contains supplementary material, which is available to authorized users.

## Background

Tumor samples that are obtained temporally or spatially from a cancer patient are heterogeneous in nature [1, 2]. These samples contain genetically diverse sub-population of cells often referred to as tumor subclones [1, 3, 4]. Each subclone harbors a distinct mutational profile that uniquely characterizes the genome of the cells in that particular subclone [5–7]. Mutational and evolutionary processes that drive tumor progression are partly responsible for the observed genetic differences that distinguish these subclones. For instance, somatic variations among the subclones are as a result of mutations that are acquired by chance in the cell during tumor progression [8, 9].

The advancements in high-throughput sequencing technologies over the last decade [1, 10] have put a searchlight on studies that are related to tumor heterogeneity. For instance, some methods concentrate on probing individual cell using fluorescent markers [11, 12] while others employ single cell sequencing [13–16]. However, these approaches have their downsides. As an example, the use of single cell sequencing to probe large number of cells remains too expensive. On the other hand, methods like whole genome sequencing (WGS) and whole exome sequencing (WES) of tumor samples allow for proper and adequate quantification of somatic mutations in the cells [17].

One way to resolve tumor heterogeneity is to computationally characterize and identify the tumor subclones in the samples, employing the datasets from WGS and WES. Generally, computional approach at resolving tumor heterogeneity is a very challenging task [18]. It involves an estimation of the distinct single nucleotide variant (SNV) profiles/genotypes and their respective proportions in the samples. The result from such task assists in the design of effective therapy in combating cancer, aids correct cancer prognosis [19] and minimizes chemotherapy resistance [20].

In the literature, various computational methods have been proposed to resolve tumor heterogeneity [18]. Most prominent among these methods model the SNV profiles/genotypes of subclones with a binary matrix. Each row of the genotype matrix corresponds to a locus/SNV and each column represents the SNV profile of a subclone. Further, computational approach can be viewed as either an indirect or a direct estimation problem, depending on how the genotype matrix is obtained. In the former, genotypes of subclones in the tumor samples are not directly inferred. Rather mutations with similar cellular prevalence are first grouped as mutation clusters. As a result, further analyses are often required in order to obtain the genotypes/SNV profiles of tumor subclones in the samples [21–25].

The direct approach employs the feature allocation model for the decomposition of the observed variant allele fractions (VAFs) into matrices of genotypes (**Z**) and proportions (**W**) [26–29]. In addition to the VAF dataset, some methods include copy number information in the analysis of tumor heterogeneity [24]. These methods simultaneously model the copy number variation and SNV datasets. A host of methods under the direct approach assume a fixed number of subclones, and model the genotypes of subclones with a binary matrix. Each column of the matrix corresponds to the SNV profile of a subclone: 0 and 1 denoting the absence and presence of a particular SNV in a subclone [26, 27]. However, in reality, the exact number of subclones is not known prior to the analysis of the samples. To estimate model parameters of the feature allocation model, [27] proposed an expectation-maximization (EM) algorithm [30] that returns point estimates of model parameters. Markov chain Monte Carlo (MCMC) [31, 32], which has been the gold standard algorithm in the literature [21, 24, 26, 28], returns point estimates and variabilities of model parameters. As noted in [26, 28], when the number of SNVs is large, MCMC algorithm is plagued with computational issues. With EM and MCMC algorithms, whenever more VAFs are available from newly called SNV(s), there is no provision for improvement of the existing parameter estimates with the new datasets.

In this paper, we propose a state-space formulation of the feature allocation modeling framework. Our work also describes a sequential Monte Carlo (SMC) algorithm [33, 34] for inferring all the unknown model parameters that explain tumor heterogeneity. These parameters include the binary matrix of genotypes and the proportions of tumor subclones in the samples. In particular, our state-space formulation considers the sequential construction of the binary genotype matrix by making use of Indian buffet process (IBP) [35–37]. IBP describes the prior distribution of a binary matrix with a fixed number of rows and an unknown number of columns. Other parameters of the feature allocation model, including the proportions of tumor subclones, are considered as the parameters of our state-space model. The observed VAF, which is the input data, is processed rowwise: this enables scalability to any number of rows. In the SMC framework, observed measurements are processed one at a time. At every instance of time, the posterior probability density function (PDF) of the state at that time is computed via approximation [38–41]. With extensive simulation, we compare SeqClone with other computational methods for resolving tumor heterogeneity. Overall, in terms of accuracy of the estimates of model parameters, SeqClone demonstrates comparable and sometimes superior performance to other methods.

The remainder of this paper is organized as follows. In the “Results” section, we investigate the performance of SeqClone, using simulated datasets and chronic lymphocytic leukemia (CLL) datasets, the real tumor samples obtained from three patients in [42]. In the “Discussion” section, we discuss the results obtained from the proposed algorithm. “Conclusions” section concludes the paper. Finally, the “Method” section details the description of system model and problem formulation.

## Results

In this section, we report the performance of the proposed algorithm using simulated and real tumor datasets. We compared model estimates, matrices of genotypes and proportions, from the proposed algorithm to those obtained from other similar algorithms. In real tumor datasets, similar to the manual approach considered in [27], we hypothesized phylogenetic trees from the estimated matrix of genotypes. Particularly, we assumed that the set of mutations that are grouped together in a tumor subclone comprises of: all the mutations that belong to its ancestors on the tree and the mutations on the edge that connect the subclone to its parent subclone. With this simple rule, we were able to construct the possible phylogenetic trees that are consistent with the estimated matrix of genotypes. For the simulation experiments, we employed a reverse of the above rule to generate binary genotype matrices from phylogenetic trees. Finally, we compared the runtimes of the different algorithms for subclone inference.

### Simulated datasets

We generated datasets for average sequencing depth *r*∈{50,200,1000} per locus, number of tumor subclones *C*∈{3,4,5}, number of tumor samples *S*∈{3,4,...,10} and number of genomic loci *T*∈{20,40,60,80,100,5000}. For a given number of tumor subclones *C* and number of genomic loci *T*, we simulated a phylogenetic tree from where the genotype matrix **Z** is obtained. For the phylogenetic tree simulation, we grouped the *T* mutations into *C* subclones uniformly at random. The mutations in each subclone are assumed to first appear in that particular subclone on the tree. One of the subclones is randomly selected as the root node and the rest *C*−1 subclones are iteratively connected to the tree. Specifically, an unattached subclone (child) and a parent subclone on the tree are randomly selected. The child subclone is attached to the parent subclone and the new set of mutations in the child subclone is a union of the mutations in the parent and the mutations in the child subclone. The mutational profiles of the subclones are the columns of the genotype matrix **Z**.

Given the genotype matrix, along with specific values of *r* and *S*, we generated the input data to the proposed algorithm, i.e., the matrices of variant count **Y** and total count **V**. We generated each entry of **V**, i.e., *v*_*ts*_ from Pois(*r*). We generated each entry of **Y**, i.e., *y*_*ts*_ as follows: sampled each column of the proportion matrix **W** independently from Dir([*a*_0_,*a*_1_,...,*a*_4_]) (*a*_0_=0.2 and *a*_*c*_, *c*∈{1,...,4} randomly chosen from the set {2,4,5,6,7,8}), defined *p*=0.02, computed *p*_*ts*_ following (2) in the “Method” section, and sampled *y*_*ts*_ from binomial(*v*_*ts*_,*p*_*ts*_).

The proposed algorithm, Clomial [27], BayClone [28], and Cloe [26] were run on the simulated datasets. We defined the following metrics to quantify the estimation strength of the algorithms: genotype error (*e*_*Z*_), proportion error (*e*_*W*_) and success probabilities error ($e_{p_{ts}}$). Mathematically, these errors are defined as

$$ \begin{aligned} e_{Z} \,=\, \frac{1}{TC} \sum\limits_{t = 1}^{T} \sum\limits_{c = 1}^{C} \left| \hat{z}_{tc} \,-\, z_{tc} \right|, \hspace{1mm} e_{W} = \frac{1}{CS} \sum\limits_{c = 0}^{C} \sum\limits_{s = 1}^{S} \left| \hat{w}_{cs} - w_{cs} \right|, \end{aligned} $$

and

$$ \begin{aligned} e_{p_{ts}} \,=\, \frac{1}{TS} \sum\limits_{t= 1}^{T} \sum\limits_{s = 1}^{S} \left| \hat{p}_{ts} \,-\, p_{ts} \right|, \hspace{1mm} \text{where} \hspace{1mm} \hat{p}_{ts} \,=\, \hat{p} \hat{w}_{0s} \,+\, \sum\limits_{c = 1}^{C} \hat{z}_{tc} \hat{w}_{cs}. \end{aligned} $$

The problem of estimating genotype matrix and proportions matrix is a blind decomposition problem. This implies that after the analysis, we are unaware of the columns of the estimated genotype matrix that correspond to the columns of the true genotype matrix. We resolved this by computing the genotype error with every permutation of the columns of the estimated genotype matrix. We selected the permutation that resulted in the smallest error and we used the selected genotype in computing the other error values. All experiments were performed on Intel(R) Xeon(R) CPU *@* 3.5GHz and a 24GB of RAM running a 64-bit Windows 7.

**Fig. 1:**
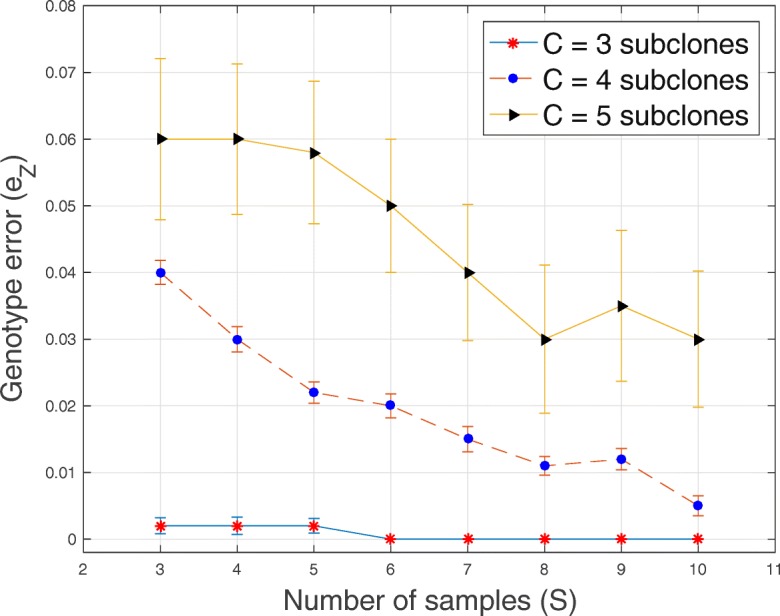
Plot of genotype error *e*_*Z*_ versus sample size *S* for *T*=20 loci, average sequencing depth *r*=1000 and *C*∈{3,4,5} subclones

**Fig. 2:**
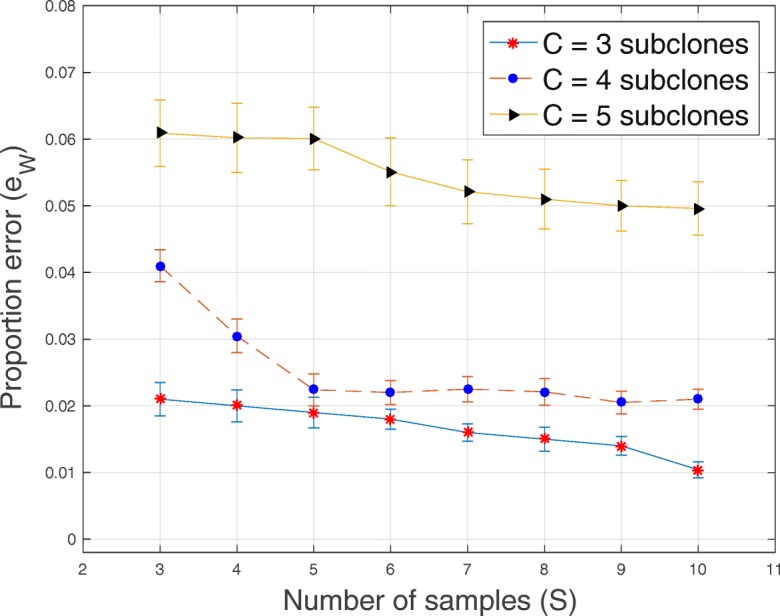
Plot of proportion error *e*_*W*_ versus sample size *S* for *T*=20 loci, average sequencing depth *r*=1000 and *C*∈{3,4,5} subclones

**Fig. 3:**
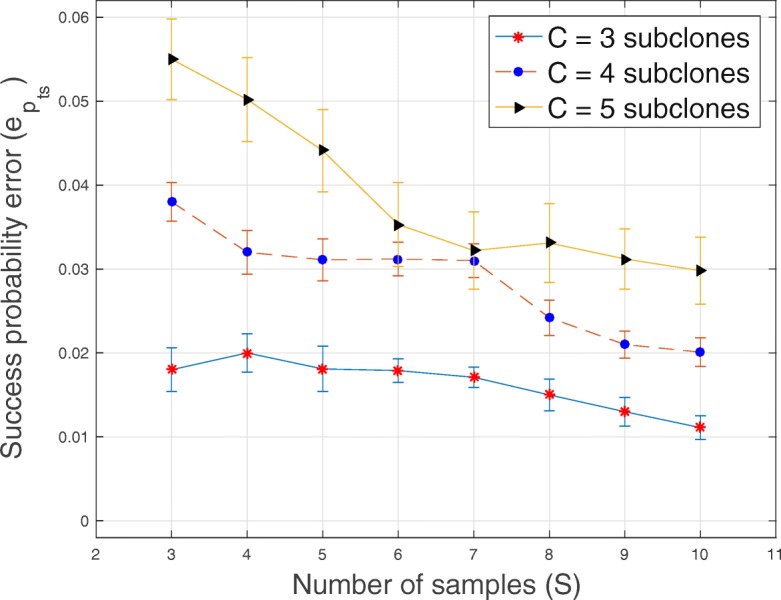
Plot of the error of success probability $e_{p_{ts}}$ versus sample size *S* for *T*=20 loci, average sequencing depth *r*=1000 and *C*∈{3,4,5} subclones

**Fig. 4:**
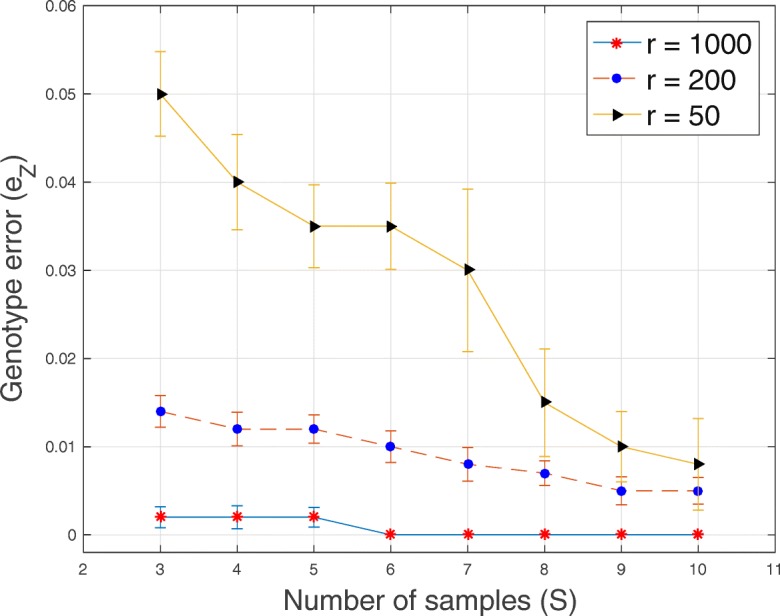
Plot of genotype error *e*_*Z*_ versus sample size *S* for *T*=20 loci, *C*=3 subclones and average sequencing depth *r*∈{50,200,1000}

**Fig. 5:**
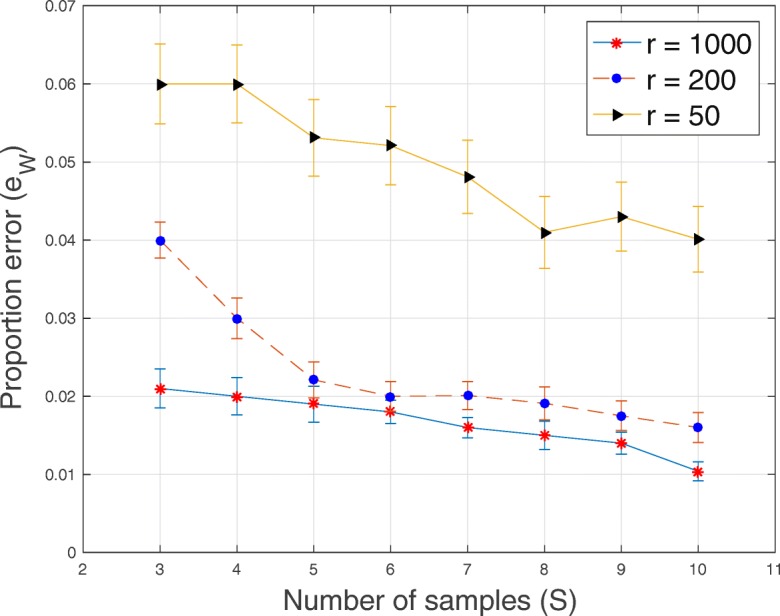
Plot of proportion error *e*_*W*_ versus sample size *S* for *T*=20 loci, *C*=3 subclones and average sequencing depth *r*∈{50,200,1000}

**Fig. 6:**
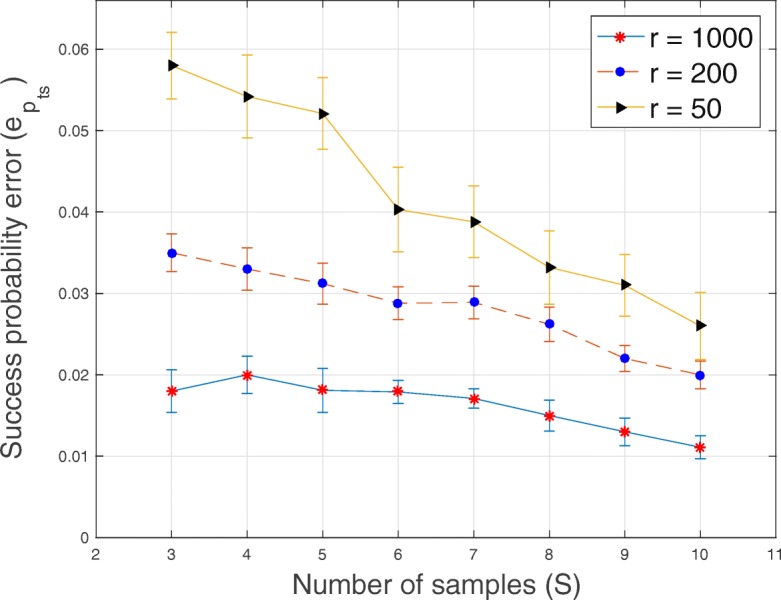
Plot of the error of success probability $e_{p_{ts}}$ versus sample size *S* for *T*=20 loci, *C*=3 subclones and average sequencing depth *r*∈{50,200,1000}

**Fig. 7:**
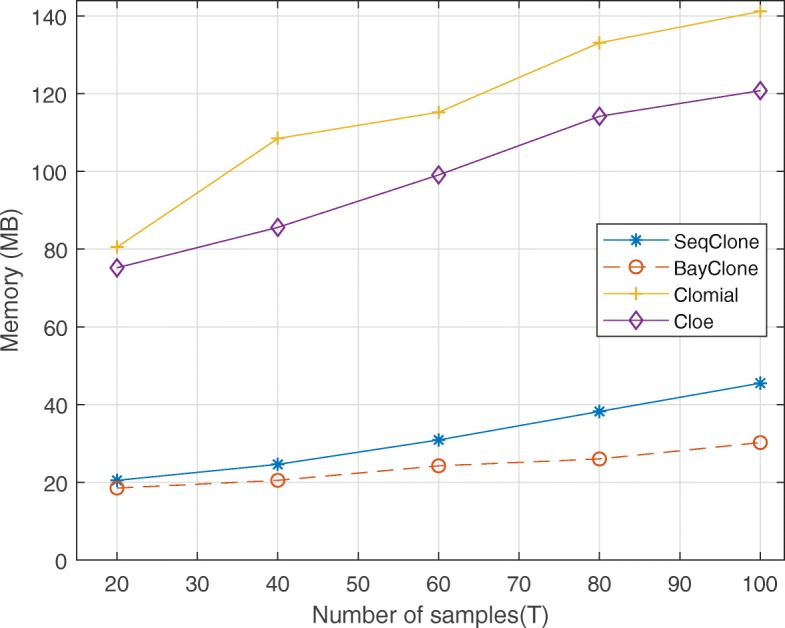
Plot of consumed memory versus number of genomic loci *T*∈{20,40,60,80,100}

In Tables 1, 2, 3 and 4 and Figs. 1, 2, 3, 4, 5, 6 and 7, we present the results obtained from analyses of simulated datasets. To compare the methods, we generated 20 datasets for every combination of number of genomic loci *T*, number of tumor subclones *C*, number of tumor samples *S* and average sequencing depth *r*. We computed the average and standard deviation of genotype error *e*_*Z*_ and proportion error *e*_*W*_ over all the 20 datasets. In Table 1, we present the average and standard deviation (in round parentheses) of the genotype and the proportion errors for all the methods when the number of loci *T*=20, number of subclones *C*=3, number of samples *S*=5 and average sequencing depth *r*∈{50,200,1000}. We excluded success probabilities error ($e_{p_{ts}}$) because not all the algorithms return an estimate of *p* in (2) (“Method” section). Similarly, in Table 2, we show, for all the methods, the average and the standard deviation of genotype and proportion errors when *T*=100, *C*=3, *S*=5 and *r*∈{50,200,1000}. The proposed algorithm demonstrates a comparable and sometimes, superior performance in terms of the accuracy of the estimated genotype and proportion matrices. It should be noted that, for BayClone, the ones in the true binary genotype matrices were changed to 0.5 before the simulation and the entries of the estimated genotype matrices greater than 0 were changed to ones before computing the errors.

**Table 1:** Genotype error (*e*_*Z*_) and proportion error (*e*_*W*_) computed for SeqClone, Clomial, BayClone and Cloe for *T*=20, *C*=3, *S*=5 and *r*∈{50,200,1000}

	SeqClone		Clomial		BayClone		Cloe	
*r*	*e*_*Z*_	*e*_*W*_	*e*_*Z*_	*e*_*W*_	*e*_*Z*_	*e*_*W*_	*e*_*Z*_	*e*_*W*_
50	0.035 (0.005)	0.053 (0.005)	0.040 (0.005)	0.071 (0.005)	0.080 (0.007)	0.059 (0.006)	0.065 (0.005)	0.064 (0.008)
200	0.012 (0.002)	0.022 (0.002)	0.025 (0.004)	0.046 (0.007)	0.075 (0.009)	0.062 (0.008)	0.060 (0.006)	0.052 (0.003)
1000	0.002 (0.001)	0.019 (0.002)	0.020 (0.002)	0.039 (0.004)	0.060 (0.004)	0.038 (0.005)	0.065 (0.004)	0.037 (0.004)

**Table 2:** Genotype error (*e*_*Z*_) and proportion error (*e*_*W*_) computed for SeqClone, Clomial, BayClone and Cloe for *T*=100, *C*=3, *S*=5 and *r*∈{50,200,1000}

	SeqClone		Clomial		BayClone		Cloe	
*r*	*e*_*Z*_	*e*_*W*_	*e*_*Z*_	*e*_*W*_	*e*_*Z*_	*e*_*W*_	*e*_*Z*_	*e*_*W*_
50	0.030 (0.004)	0.023 (0.003)	0.055 (0.007)	0.094 (0.006)	0.078 (0.007)	0.059 (0.006)	0.041 (0.005)	0.064 (0.008)
200	0.015 (0.003)	0.014 (0.001)	0.050 (0.006)	0.050 (0.006)	0.080 (0.006)	0.061 (0.006)	0.080 (0.005)	0.081 (0.004)
1000	0.004 (0.001)	0.011 (0.001)	0.045 (0.004)	0.051 (0.005)	0.070 (0.006)	0.055 (0.005)	0.070 (0.005)	0.066 (0.005)

**Table 3:** Average and standard deviation of $e_{p_{ts}}$, *e*_*Z*_ and *e*_*W*_ for *T*∈{100,5000}, *S*=5, *C*=3, and *r*=1000

	*e*_*Z*_	*e*_*W*_	$e_{p_{ts}}$
100	0.002 [0.000]	0.010 [0.002]	0.014 [0.001]
5000	0.002 [0.001]	0.004 [0.001]	0.009 [0.002]

Average and standard deviation are taken of 20 datasets

**Table 4:** Runtimes, *e*_*Z*_ and *e*_*W*_ for *T*=20, *S*=5, *C*=3, and *r*=1000

Error	SeqClone	Clomial	BayClone	Cloe
	55 min	53 min	93 min	101 min
*e*_*Z*_	0.005	0.015	0.050	0.060
*e*_*W*_	0.018	0.034	0.036	0.035

In Figs. 1, 2, 3, 4, 5 and 6, for SeqClone, we present the errorbar plots for the average and standard deviation over 20 datasets for different combinations of the number of loci, sample size, number of subclones and average sequencing depth. The standard deviation is the vertical line above and below the average value in the errorbar plots. Figures 1, 2 and 3 show how the errors vary across different sample sizes for different subclones. There is an improvement, for all the subclones, in the estimates of all model parameters when the number of tumor samples increases. Similarly, in Figs. 4, 5 and 6, estimates of model parameters improves when the average sequencing depth increases. In the first row in Table 3, we present, for SeqClone, the result of the permutations of rows of the input data. For the dataset with *T* = 100,*C* = 3,*r* = 1000 and *S*=5, we ran SeqClone with randomly selected 100 permutations of the rows of the input data matrices and we computed the average and standard deviation of the errors (row one in Table 3). In row two in Table 3, we present results for higher number of genomic loci. In particular, we present the average and standard deviation of errors over 20 runs for the datasets with *T*=5000, *C*=3, *r*=1000 and *S*=5.

Lastly, we present the runtimes and memory consumption for all the methods when performing a section of the experiments in Table 1. For the proposed algorithm, we ran the algorithm 20 times with 1000 particles. For the MCMC-based algorithms (Cloe and BayClone), we ran 30,000 chains. For Clomial, we ran 2000 iterations. The runtimes for all the methods on a 3.5Ghz Intel 8 cores running MATLAB and the associated genotype and proportion errors for the dataset from *T*=20, *C*=3, *r*=1000, and *S*=5 are in Table 4. In addition, for this particular dataset, we report the estimated sample mean and sample standard deviation of the relative frequency of variant reads that are produced as error (parameter *p* in “Method” section) from SeqClone and BayClone. For SeqClone, the mean is 0.019 and the standard deviation is 0.0012. Likewise, for BayClone, the mean is 0.022 and the standard deviation is 0.0011. In Fig. 7, we present the memory consumption by all the algorithms for different genomic loci (*T*∈{20,40,60,80,100}). In general, Clomial is the most memory efficient of all the algorithms. However, SeqClone consumes lesser memory when compared to BayClone and Cloe.

### Real biological tumor samples

Next, we present the results obtained from applying the proposed algorithm to real biological tumor datasets. Particularly, we analyzed the datasets of three patients with B-cell CLL namely: CLL077, CLL006, and CLL003 [42]. Complete datasets and the data pre-processing steps are in [42]. In Additional file 1, we include the analysis results with Clomial, BayClone and Cloe.

#### CLL077:

**Fig. 8:**
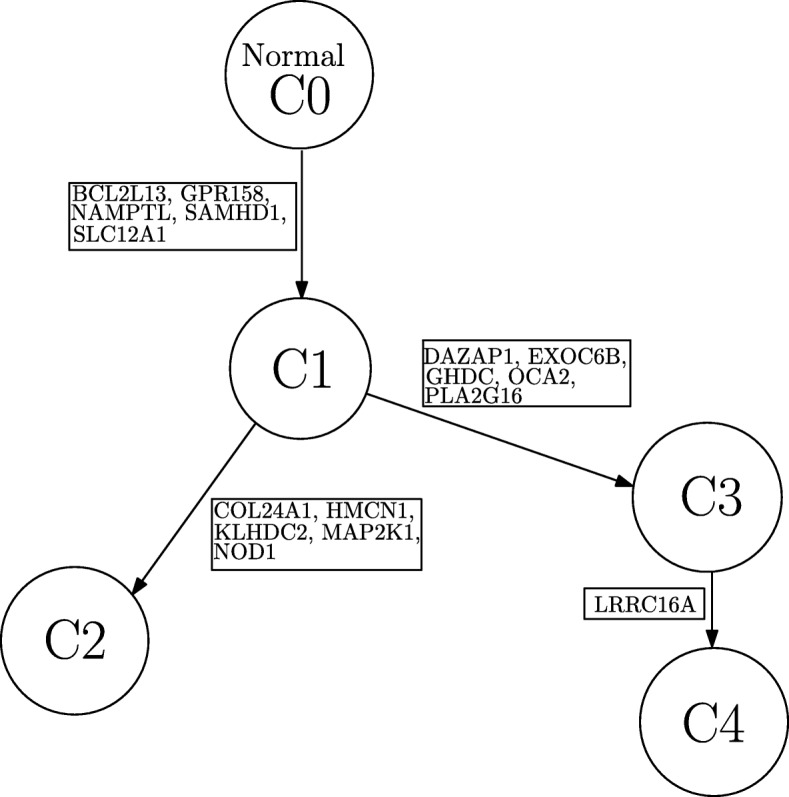
Phylogenetic tree from CLL077

Here, we present the results obtained from analyzing the dataset from patient CLL077 with SeqClone. This dataset had 16 distinct loci probed for tumor heterogeneity. These are shown in the first row in Table 5. We present our analysis results in the main paper, and the estimates for other methods in Additional file 1. In concordance with other methods, SeqClone estimated 4 subclones as shown in Table 5, and also produced SNV profiles that are similar to those obtained from the three other methods. Also, the proportions of tumor subclones exhibit similar trend in all the 5 tumor samples across various methods. For instance, the abundance of sublone 1 in sample ‘a’ in Clomial, BayClone, Cloe and SeqClone are 0.27,0.21,0.16 and 0.27, respectively. This trend continues in all other samples except in sample ‘e’ where Clomial deviates from this normal trend, i.e., Clomial, BayClone, Cloe and SeqClone are 0.43,0.07,0.03, and 0.16, respectively. On this dataset, SeqClone produced a consistent result with other methods in estimating the SNV profiles of subclones and their proportions in all the samples (Tables 5, 6 and in Additional file 1). The constructed phylogenetic tree from the SNV profiles for CLL077 is shown Fig. 8.

**Table 5:** *CLL077*: estimate of genotype matrix/mutational profile

Gene	BCL2L13	COL24A1	DAZAP1	EXOC6B	GHDC	GPR158	HMCN1	KLHDC2	LRRC16A	MAP2K1	NAMPT	NOD1	OCA2	PLA2G16	SAMHD1	SLC12A1
C1	1	0	0	0	0	1	0	0	0	0	1	0	0	0	1	1
C2	1	1	0	0	0	1	1	1	0	1	1	1	0	0	1	1
C3	1	0	1	1	1	1	0	0	0	0	1	0	1	1	1	1
C4	1	0	1	1	1	1	0	0	1	0	1	0	1	1	1	1

**Table 6:** CLL077: estimate of the proportions of subclones in the samples

Subclone	a	b	c	d	e
C0	0.00	0.00	0.00	0.05	0.35
C1	0.27	0.15	0.14	0.18	0.16
C2	0.02	0.04	0.05	0.13	0.28
C3	0.35	0.29	0.41	0.30	0.12
C4	0.36	0.52	0.40	0.34	0.09

#### CLL006:

**Fig. 9:**
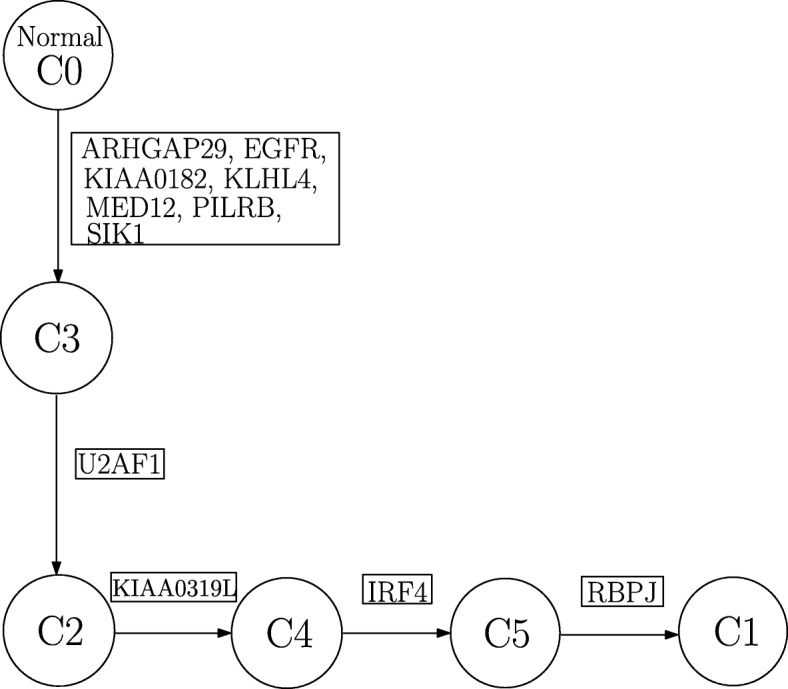
Phylogenetic tree from CLL006

This dataset comprises of 11 genomic loci. These are shown in the first row in Table 7. We analyzed the dataset with SeqClone, and the estimates of genotype and proportions matrices are in Tables 7 and 8. The constructed phylogenetic tree is shown Fig. 9. SeqClone and BayClone estimated 5 distinct subclones, Clomial had 4 subclones and Cloe recovered 6 subclones. Details of the estimates from Clomial, BayClone and Cloe are in Additional file 1.

**Table 7:** CLL006: estimate of genotype matrix/mutational profile

Gene	ARHGAP29	EGFR	IRF4	KIAA0182	KIAA0319L	KLHL4	MED12	PILRB	RBPJ	SIK1	U2AF1
C1	1	1	1	1	1	1	1	1	1	1	1
C2	1	1	0	1	0	1	1	1	0	1	1
C3	1	1	0	1	0	1	1	1	0	1	0
C4	1	1	0	1	1	1	1	1	0	1	1
C5	1	1	1	1	1	1	1	1	0	1	1

**Table 8:** CLL006: estimate of the proportions of subclones in the samples

Subclone	a	b	c	d	e
C0	0.00	0.00	0.00	0.00	0.00
C1	0.10	0.19	0.07	0.19	0.21
C2	0.41	0.09	0.19	0.18	0.17
C3	0.23	0.24	0.30	0.16	0.08
C4	0.09	0.21	0.19	0.17	0.27
C5	0.17	0.27	0.25	0.30	0.27

#### CLL003:

**Fig. 10:**
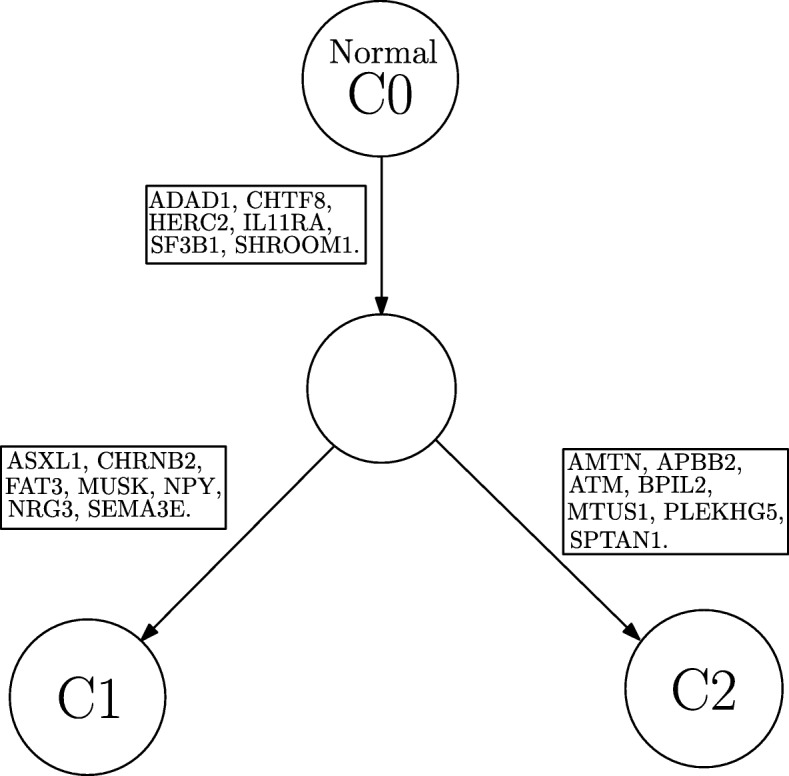
Phylogenetic tree from CLL003

The dataset from patient CLL003 has 20 distinct genomic loci. This is shown in the first row in Table 9. In this dataset, Clomial and Cloe produced 2 distinct subclones with considerably high proportions in the samples and 2 others with very small proportions across all samples. SeqClone and BayClone estimated the first 2 major subclones that dominate the 5 samples with proportions shown in Table 10 (and Additional file 1: Table S6). The constructed phylogenetic tree for CLL003 is shown in Fig. 10.

**Table 9:** *CLL003*: estimate of genotype matrix/mutational profile

Gene	ADAD1	AMTN	APBB2	ASXL1	ATM	BPIL2	CHRNB2	CHTF8	FAT3	HERC2	IL11RA	MTUS1	MUSK	NPY	NRG3	PLEKHG5	SEMA3E	SF3B1	SHROOM1	SPTAN1
C1	1	0	0	1	0	0	1	1	1	1	1	0	1	1	1	0	1	1	1	0
C2	1	1	1	0	1	1	0	1	0	1	1	1	0	0	0	1	0	1	1	1

**Table 10:** CLL003: estimate of the proportions of subclones in the samples

Subclone	a	b	c	d	e
C0	0.00	0.00	0.35	0.00	0.01
C1	0.08	0.05	0.53	0.99	0.98
C2	0.92	0.95	0.12	0.01	0.01

Finally, we investigated the behavior of the algorithms in terms of runtime and memory consumption, when applied to simulated and real datasets of similar size: *T*=20 and *S*=5. We present the results in Table 11. Runtimes (without parentheses) are in minutes and the consumed memory (in round parentheses) are in MB.

**Table 11:** Runtimes and memory consumption for simulated and real biological dataset

	SeqClone	Clomial	BayClone	Cloe
Simulated data	55 (20.48)	53 (18.50)	93 (80.52)	101 (75.20)
CLL003	57 (20.60)	54 (18.80)	98 (81.00)	102 (75.50)

## Discussion

Tumor heterogeneity describes a situation where bulk tumor samples have numerous subpopulations of cancer cells and each subpopulation has unique features that distinguish it from other subpopulations in the samples. It has been recognized as the major cause of relapse in cancer patients. One way to resolve tumor heterogeneity is by deconvolving the VAFs data from the next-generation sequencing to the genotypes and the proportions of subpopulations of cancer cells in the samples. In this paper, to resolve tumor heterogeneity, we interpreted the VAFs data using the feature allocation model [27, 28].

We developed the feature allocation model into a state-space framework so that VAFs with large number of genomic loci can be adequately modeled. We proposed a sequential algorithm, SeqClone, to infer all the parameters of our state-state model. The inferred parameters, which describe tumor heterogeneity, include: the genotypes of all the genomic loci in every subpopulation and their respective proportions in the tumor samples. With the state-space modeling framework and the sequential algorithm, computational problem that is often encountered by other methods for interpreting tumor heterogneity in the presence of large genomic loci is eliminated [26, 28]. It should be noted that, in this work, like some previous methods [27, 43], only somatic SNVs/mutations are modeled and we assume that these mutations are unaffected by copy number aberrations or rearrangements in the cancer genome. With this modeling assumption, extreme care must be taken when using SeqClone to interpret tumor heterogeneity.

In the “Results” section, we presented the results from running SeqClone and three other algorithms: Clomial, BayClone and Cloe, on simulated and real cancer datasets. For the simulation experiments, we generated several simulated datasets and compared the results from all the algorithms. SeqClone produced comparable, and sometimes better performance in the estimation of model parameters. On the real cancer datasets ([42]), SeqClone produced satisfying results that are comparable to other methods.

Also, because of the sequential nature by which the VAFs are processed by SeqClone, VAFs from previously unprobed genomic loci can be analyzed to improve the existing results, a feature that is absent in other algorithms.

## Conclusions

Finally, we have demonstrated the efficacy of sequential Monte Carlo algorithm in the analysis of VAFs datasets that are obtained from heterogeneous tumor samples. The proposed method does not assume that the number of subclones is known/fixed prior to analysis and this allows the ‘correct’ number of subclones to be estimated from the tumor samples. Also, because of the sequential nature by which the proposed algorithm handles the VAFs datasets, the analysis can easily be scaled to a very large dataset. In addition, the current framework can be extended to a more general case that involves the estimation of mutation and the copy number profiles of the tumor subclones that are present in the tumor samples.

## Method

### System model and problem formulation

Before going to the details of our modeling approach, we define all the mathematical notations that are used in this paper. *p*(·) denotes a PDF, *p*(·|·) denotes a conditional PDF, *P*(·) denotes a probability mass function (PMF) and *P*(·|·) denotes a conditional PMF. Likewise, binomial(*n*,*p*) denotes a binomial distribution with *n* exact number of trials and *p* probability of success at each trial. Bern(*p*) denotes a Bernoulli distribution with success probability *p* and $\mathcal {N}\left (\mu,\sigma ^{2}\right)$ denotes a univariate Gaussian distribution with mean *μ* and variance *σ*^2^. Also, gamma(*α*_0_,*β*_0_) denotes a gamma distribution (*α*_0_ is the shape parameter and *β*_0_ is the rate parameter) and beta(*α*_1_,*β*_1_) denotes a beta distribution where *α*_1_ and *β*_1_ are the shape parameters. Pois(*λ*) denotes a Poisson distribution with mean parameter *λ* and Dir(***α***) denotes a Dirichlet distribution with a vector of concentration parameters ***α***. Lastly, *Γ*(·) denotes the gamma function and $\hat {x}$ denotes the estimate of variable *x*.

Two important quantities that are obtained from WGS and WES of tumor samples are the variant count and total count at each of the probed genomic locus. We denote the matrix of variant count by **Y** and the matrix of total count by **V**. Each of the matrices has a dimension *T*×*S*, where *T* is the number of genomic loci/SNVs and *S* is the total number of tumor samples. We denote the number of reads that bear the variant count at locus *t* in sample *s* as *y*_*ts*_. Likewise, we denote the total number of reads at locus *t* in sample *s* as *v*_*ts*_. In our formulation, we assume that the genomic loci are unaffected by copy number aberrations or rearrangement of the cancer genome [27, 43]. We employ the binomial sampling model [27, 28] in modeling the input data matrices, given as

1 $$ \begin{aligned} y_{ts} \stackrel{{ind.}}{\sim} \text{binomial}\left(v_{ts},p_{ts}\right), \hspace{2mm} t = 1,...,T, \hspace{1mm} s = 1,...,S, \end{aligned}  $$

*p*_*ts*_, *t*=1,...,*T*, *s*=1,...,*S* are the success probabilities defined as [28]

2 $$ \begin{aligned} p_{ts} = w_{0s}p + \frac{1}{2}\sum\limits_{c = 1}^{C} z_{tc}w_{cs}, \end{aligned}  $$

where *z*_*tc*_, a binary variable, represents the two possible states of an allelic genotype at locus *t* in subclone *c* and *C* represents the number of tumor subclones, an unknown variable. Under this framework, if *z*_*tc*_=1, it implies that locus *t* in subclone *c* has reads that bear variant sequence. Likewise, if *z*_*tc*_=0, there are no reads that bear variant sequence at that locus. We assume that if a mutation is present in a particular subclone, then at that genomic locus, the subclone is heterozygous with copy number equal to one.

The term ${\sum }_{c=1}^{C} z_{tc}w_{cs}$ in (2) defines *p*_*ts*_ as a weighted sum of effects of an unknown number of subclones in the tumor samples. Also, effects of experimental and data processing noises are captured by *w*_0*s*_*p* in (2). In particular, *p* is the relative frequency of variant reads that are generated as a result of error during upstream data analysis [28]. For *t*=1,...,*T*, *s*=1,...,*S*, we can write (2) as

3 $$ \begin{aligned} \mathbf{P}_{ts} = \mathbf{Z}^{\prime} \cdot \mathbf{W}, \end{aligned}  $$

with $\mathbf {Z}^{\prime } = \left [\mathbf {p} \hspace {1mm} \frac {1}{2}\mathbf {Z}\right ]$. **P**_*ts*_ is a *T*×*S* matrix of success probabilities, **Z** is a *T*×*C* binary matrix and **p** is a column vector with all its elements equal to *p*.

Each column of matrix **Z** represents the SNV profile of a tumor subclone and each column of matrix **W** represents the proportions of subclones in a sample. Thus, **Z**, **W**, *C* and *p* explain the inherent heterogeneity in the tumor samples. We perform a joint inference on all these variables by formulating the system model in a state-space framework and then derive an SMC algorithm to infer all the model parameters.

### State-space formulation

Here, we describe the state transition and the observation models of our state-space formulation of the feature allocation model (solution to (3)). Before going through the details of our formulation, we will briefly describe the prior distribution on a left-ordered binary matrix **Z** that has a finite number of rows and an unknown number of columns [35, 36]. By left-ordered, we mean that the columns of the binary matrix are ordered from left to right according to the magnitude of the binary in the columns and the first row is considered the most significant. Mathematically, the distribution is expressed as

4 $$ {{}\begin{aligned} P(\mathbf{Z}) = \frac{\alpha^{C_{+}}}{\prod_{h=1}^{2^{T}-1}C_{h}!} \exp \left\{ -\alpha H_{T} \right\} \prod\limits_{c=1}^{C^{+}} \frac{ \left(T - m_{c}\right)! \left(m_{c} - 1\right)!}{T!}, \end{aligned}}  $$

where *m*_*c*_ represents the number of non-zero entries in the *c*^*th*^ column of matrix **Z**, *T* represents the finite number of rows in matrix **Z**, *C*_+_ represents the number of columns in matrix **Z** that do not sum to zero. $H_{T} = {\sum }_{t = 1}^{T} 1/t$ represents the *T*^*th*^ harmonic number and *C*_*h*_ represents the number of columns in matrix **Z** that form a sequence of ones and zeros corresponding to the binary representation of the number *h* when read top-to-bottom.

Fortunately, the prior distribution described in (4) can be viewed as the outcome of IBP, a sequential generative process for the binary matrix. Given that in an Indian buffet restaurant, we have *T* customers who come into the restaurant one after the other. Assume that the first customer comes into the restaurant and fills her plate from the first *c*_1_=Pois(*α*) distinct dishes. Then the *t*^*th*^ customer chooses a particular dish with probability *m*_*c*_/*t*, *m*_*c*_ being the number of people that have chosen the *c*^*th*^ dish before her, and in addition, she adds Pois(*α*/*t*) new dishes. Following the dish serving rule, if we record the choices of the *T* customers on the different dishes as a binary matrix such that an entry is one if the customer chose the dish and zero otherwise, such a matrix is a draw from the distribution in (4) [36]. The IBP process is a sequential process in such a way that the choices of the *t*^*th*^ customer are only dependent on the customers that were in the restaurant before her.

In our state-space framework, we designate tumor subclones as the dishes, the genomic loci as the customers and the *t*^*th*^ customer as the observation at *time* *t* (*t*^*th*^ row of the input data). We write **z**_*t*_=[*z*_*t*1_,*z*_*t*2_,...,*z*_*tC*_], the *t*^*th*^ row of **Z** as the state at time *t*. Thus, according to the sequential process described by the IBP, our state transition model is written as

5 $$ \begin{aligned} P\left(\mathbf{z}_{t}|\mathbf{Z}_{t-1},\alpha\right), \end{aligned}  $$

where **Z**_*t*−1_ represents a binary sub-matrix of the top *t*−1 rows in **Z**. We present the algorithm to draw a sample from (5) in **Algorithm 1**. In the algorithm, **Z**_*t*_ is obtained from **Z**_*t*−1_ and if in the process, additional non-zero column(s) is/are created in **Z**_*t*_, i.e., Pois(*α*/*t*)>0, then additional row(s) will also be added to matrix **W**. We re-parameterize matrix **W** to easily accommodate any possible change in its dimension by writing $w_{cs} = \theta _{cs}/{\sum }_{c^{\prime }= 0}^{C} \theta _{c^{\prime }s}$. In other word, we estimate *θ*_*cs*_ instead of *w*_*cs*_ and we compute *w*_*cs*_ from the estimates of *θ*_*cs*_.


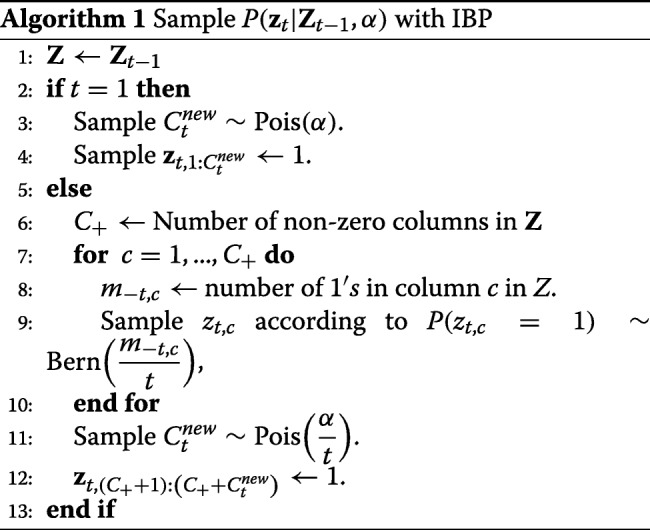


For all the parameters of our state-space model, i.e., matrix **W** and the relative frequency of variant reads *p*, we employ random walk model to create artificial dynamics

6 $$ \begin{aligned} \phi_{t} \sim p\left(\phi_{t}|\phi_{t-1}\right) = \mathcal{N}\left(\phi_{t-1},\sigma^{2}\right), \\ \phi_{t} \in \left\{ p, \theta_{cs}, c = 0,1,...,C, s = 1,...,S\right\}. \end{aligned}  $$

Thus, (5)-(6) describe the system state transition of our state-space framework.

The observation model that describes the measurement at time *t* in the state-space framework is given by

7 $$ \begin{aligned} \mathbf{y}_{t} \sim P\left(\mathbf{y}_{t}|\mathbf{Z}_{1:t},\mathbf{W},p\right) &= P(\mathbf{y}_{t}|\mathbf{z}_{t},\mathbf{W},p) \\ & = \prod\limits_{s=1}^{S}\text{binomial}\left(y_{ts}|v_{ts},p_{ts}\right), \end{aligned}  $$

where **y**_*t*_ represents the measurement at time *t*, the *t*^*th*^ row of **Y**. Note that, given the state value at time *t* **z**_*t*_), the measurement at this time-step is conditionally independent of all the past measurements **Y**_*t*−1_. Thus, (7) details the observation model for the proposed state-space framework. Finally, (5) - (7) state the proposed state-space framework, comprising of the state transition and the observation models for resolving tumor heterogeneity. In summary, the framework described considers, at time *t*, the *t*^*th*^ row of the input data matrices (**Y** and **V**) as the observed measurement at *time t*. The *t*^*th*^ row of the binary genotype matrix **Z** is treated as the hidden state at *time t*. The proportions **W** and the relative frequency *p* are treated as the parameters of the model.

### The SMC algorithm

Here, we present a brief description of the SMC filtering approach [33, 34] to make inference on the states (matrix **Z**) and the parameters (matrix **W** and *p*) of the proposed state-space framework. Assume that we have a dynamic system which has a hidden state variable **x**_*t*_, a measurement variable **y**_*t*_, an initial state model (state model when *t*=0) and a state transition model for other time-steps (∀*t*>0). In this paper, **x**_*t*_ comprises of two types of variables: continuous variables *ϕ*_*t*_, $\phi _{t} \in \left \{ p_{0}^{t}, \theta _{cs}^{t}, c = 0,1,...,C, s = 1,...,S\right \}$ and discrete variable **z**_*t*_. Also, (5) - (6) describe the state transition model and (7) describes the observation model. At every time-step, given that we have the sequence of measurements up to the present time-step, i.e., **Y**_*t*_={**y**_1_,**y**_2_,...,**y**_*t*_}, we are interested in inferring the unobserved sequence **X**_*t*_={**x**_1_,**x**_2_,...,**x**_*t*_}.

If we can obtain samples (particles) from the posterior distribution *p*(**X**_*t*_|**Y**_*t*_), then *p*(**X**_*t*_|**Y**_*t*_) can be approximated by the drawn particles. But in most cases, obtaining these particles is not viable. One way to get an estimate is by obtaining weighted particles from a different distribution *q*(**X**_*t*_|**Y**_*t*_) that has a support which incorporates the support of *p*(**X**_*t*_|**Y**_*t*_). This distribution is known as importance distribution. Given that we sample *N* times from *q*(**X**_*t*_|**Y**_*t*_), i.e., $ \left \{ \mathbf {X}_{i} \right \}_{i = 1}^{N}$, the associated weights are computed as

8 $$ {{}\begin{aligned} \tilde{w}_{t}^{i} = \frac{p\left(\mathbf{X}_{t}|\mathbf{Y}_{t}\right)}{q\left(\mathbf{X}_{t}|\mathbf{Y}_{t}\right)} \hspace{2mm} \text{and} \hspace{2mm} w_{t}^{i} = \frac{\tilde{w}_{t}^{i}}{{\sum}_{m = 1}^{N}\tilde{w}_{t}^{m}}, \hspace{1mm} i = 1,...,N. \end{aligned}}  $$

Thus, an approximation $\hat {p}\left (\mathbf {X}_{t}|\mathbf {Y}_{t}\right)$ of the original posterior distribution *p*(**X**_*t*_|**Y**_*t*_) is by

9 $$ \hat{p}\left(\mathbf{X}_{t}|\mathbf{Y}_{t}\right) \,=\, \!\sum\limits_{i = 1}^{N} w_{t}^{i} \delta\left(\mathbf{X}_{t} \,-\, \mathbf{X}_{t}^{i}\right), \hspace{1mm} \!\text{where} \hspace{1mm} \delta(\mathbf{u}) \,=\, \left\{\begin{array}{ll} \!\!1, & \text{if}\ \mathbf{u} \,=\, \underline{\mathbf{0}} \\ \!\!0, & \text{otherwise}. \end{array}\right.  $$

This procedure is termed the importance sampling theory.

Next, we describe the sequential version of the importance sampling theory. The first step is to factorize the posterior distribution of state variables at time *t*, **X**_*t*_, given all the measurements up to and including at time *t* **Y**_*t*_, i.e.,

10 $$ \begin{aligned} p\left(\mathbf{X}_{t}|\mathbf{Y}_{t}\right) &\propto p\left(\mathbf{y}_{t}|\mathbf{X}_{t},\mathbf{Y}_{t-1}\right) p\left(\mathbf{X}_{t}|\mathbf{Y}_{t-1}\right) \\ & \,=\, p\left(\mathbf{y}_{t}|\mathbf{X}_{t},\mathbf{Y}_{t-1}\right) p\left(\mathbf{x}_{t}|\mathbf{X}_{t\,-\,1},\mathbf{Y}_{t\,-\,1}\right) p\left(\mathbf{X}_{t-1}|\mathbf{Y}_{t-1}\right). \end{aligned}  $$

At time *t*, instead of sampling from the original distribution *p*(**X**_*t*_|**Y**_*t*_) to approximate *p*(**X**_*t*_|**Y**_*t*_), we obtain *N* weighted particles from the importance distribution *q*(**X**_*t*_|**Y**_*t*_). We write the importance distribution as *q*(**X**_*t*_|**Y**_*t*_)=*q*(**x**_*t*_|**X**_*t*−1_,**Y**_*t*_)*q*(**X**_*t*−1_|**Y**_*t*−1_), and we compute the associated unnormalized weights as

11 $$ \begin{aligned} \tilde{w}_{t}^{i} = \frac{p\left(\mathbf{y}_{t}|\mathbf{X}_{t}^{i},\mathbf{Y}_{t-1}\right) p\left(\mathbf{x}_{t}^{i}|\mathbf{X}_{t-1}^{i},\mathbf{Y}_{t-1}\right)}{q\left(\mathbf{x}_{t}^{i}|\mathbf{X}_{t}^{i},\mathbf{Y}_{t}\right)} \frac{p\left(\mathbf{X}_{t-1}^{i}|\mathbf{Y}_{t-1}\right)}{q\left(\mathbf{X}_{t-1}^{i}|\mathbf{Y}_{t-1}\right)}. \end{aligned}  $$

Imagine that at time *t*−1, we followed the description of the sequential version of importance sampling and we had *N* particles, $\left \{ \mathbf {X}_{t-1}^{i} \right \}_{i = 1}^{N}$, drawn from *q*(**X**_*t*−1_|**Y**_*t*−1_), and the associated normalized weights given as

12 $$ \begin{aligned} w_{t-1}^{i} \propto \frac{p\left(\mathbf{X}_{t-1}^{i}|\mathbf{Y}_{t-1}\right)}{q\left(\mathbf{X}_{t-1}^{i}|\mathbf{Y}_{t-1}\right)}, \hspace{2mm} i = 1,...,N. \end{aligned}  $$

From the weighted particles at time *t*−1, we easily obtain weighted particles at time *t*, i.e., $\left \{ \mathbf {X}_{t}^{i} \right \}_{i = 1}^{N} = \left \{ \mathbf {x}_{t}^{i},\mathbf {X}_{t-1}^{i} \right \}_{i = 1}^{N}$, where $\mathbf {x}_{t}^{i} \sim q\left (\mathbf {x}_{t}|\mathbf {X}_{t-1}^{i},\mathbf {Y}_{t}\right)$. By substituting (12) into (11), the associated unnormalized weights at time *t* satisfy the recursion

13 $$ \begin{aligned} \tilde{w}_{t}^{i} \propto w_{t-1}^{i} \frac{p\left(\mathbf{y}_{t}|\mathbf{X}_{t}^{i},\mathbf{Y}_{t-1}\right) p\left(\mathbf{x}_{t}^{i}|\mathbf{X}_{t-1}^{i},\mathbf{Y}_{t-1}\right)}{q\left(\mathbf{x}_{t}^{i}|\mathbf{X}_{t}^{i},\mathbf{Y}_{t}\right)}, \hspace{2mm} i = 1,...,N. \end{aligned}  $$

The weights are normalized to sum to one.

The optimal importance distribution that reduces variability due to one step reweighting is $ p\left (\mathbf {x}_{t}^{i}|\mathbf {X}_{t-1}^{i},\mathbf {Y}_{t}\right)$. This choice reduces the weights equation in (13) to $\tilde {w}_{t}^{i} \propto w_{t-1}^{i} p\left (\mathbf {y}_{t}|\mathbf {X}_{t-1}^{i},\mathbf {Y}_{t-1}\right)$ [44, 45]. However, we only have closed form solutions for $p\left (\mathbf {x}_{t}^{i}|\mathbf {X}_{t-1}^{i},\mathbf {Y}_{t}\right)$ and $p\left (\mathbf {y}_{t}|\mathbf {X}_{t-1}^{i},\mathbf {Y}_{t-1}\right)$ if and only if $p\left (\mathbf {y}_{t}|\mathbf {X}_{t}^{i},\mathbf {Y}_{t-1}\right)$ and $p\left (\mathbf {x}_{t}^{i}|\mathbf {X}_{t-1}^{i},\mathbf {Y}_{t-1}\right)$ are conjugates. Such conjugacy does not exist in our state-space framework. An equally efficient solution is to choose $p\left (\mathbf {x}_{t}^{i}|\mathbf {X}_{t-1}^{i}\right)$ in (5)-(6) as the importance distribution [46–49]. Because of independence assumption in the model, i.e., $p\left (\mathbf {x}_{t}^{i}|\mathbf {X}_{t-1}^{i},\mathbf {Y}_{t-1}\right) = p\left (\mathbf {x}_{t}^{i}|\mathbf {X}_{t-1}^{i}\right)$ and $p\left (\mathbf {y}_{t}|\mathbf {X}_{t}^{i},\mathbf {Y}_{t-1}\right) = p\left (\mathbf {y}_{t}|\mathbf {x}_{t}^{i}\right)$, we rewrite (13) as

14 $$ \begin{aligned} \tilde{w}_{t}^{i} & \propto w_{t-1}^{i} p\left(\mathbf{y}_{t}|\mathbf{x}_{t}^{i}\right) \\ & = w_{t-1}^{i} p\left(\mathbf{y}_{t}|\mathbf{z}_{t}^{i}, \mathbf{W}_{t}^{i}\right), \end{aligned}  $$

and then normalize the weights.

As time progresses, there is degeneracy, a condition where the variance of the weights increases [33]. To combat this, we perform resampling at every time-step [46–49]. The resampling procedure [38] is as follows : view each weight $w_{t}^{i}$ as the probability of obtaining the particle index, draw *N* particles from the probability distribution $\left \lbrace w_{t}^{i} \right \rbrace $, replace the old particles with the newly drawn particles and set the new weights to a constant value 1/*N*.

The proposed sequential algorithm, SeqClone, for estimating the states variables and the parameters of our state-space framework is highlighted in **Algorithm 2**. To initialize the algorithm, we assume the following prior distributions of the model parameters

15 $$ \begin{aligned} \theta_{0s} & \stackrel{{i.i.d}}{\sim} \text{gamma}\left(a_{0},1\right), \hspace{1mm} s = 1,...,S,\\ \theta_{cs} & \stackrel{i.i.d}{\sim} \text{gamma}\left(a_{1},1\right), \hspace{1mm} s = 1,...,S,c = 1,...,C, \hspace{1mm} \text{and}\\ p & \sim \text{beta}\left(a_{00},b_{00}\right). \end{aligned}  $$

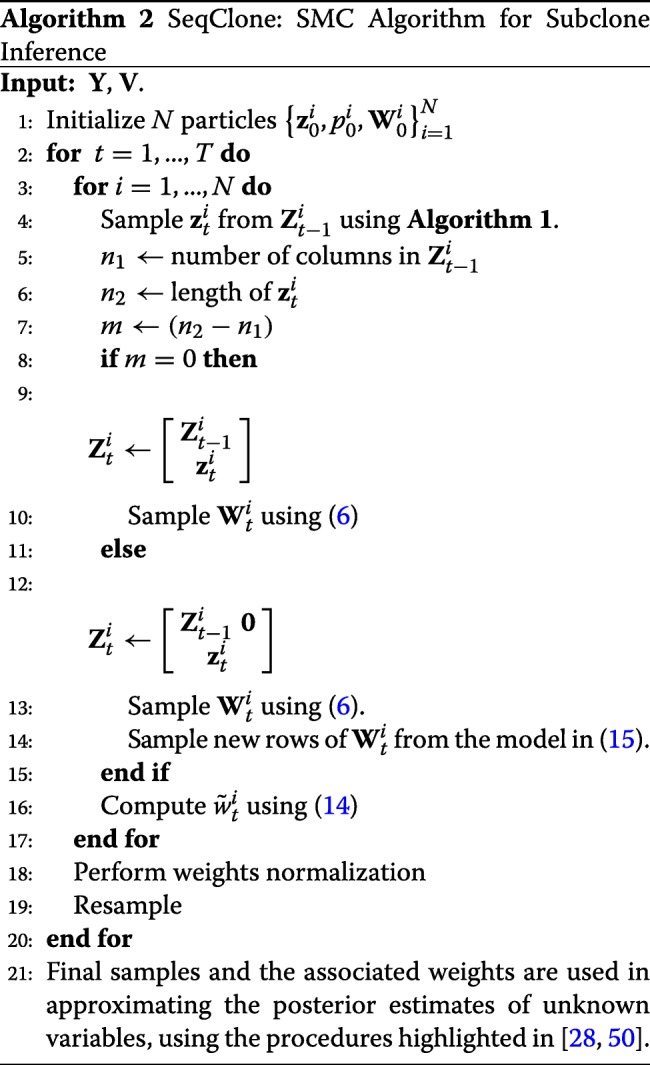


In this way, we have $w_{cs} = \theta _{cs}/{\sum }_{c^{\prime } = 0}^{C} \theta _{c^{\prime }s}$ and as a result, ${\sum }_{c^{\prime }= 0}^{C} w_{c^{\prime }s} = 1$. At every time step of the algorithm, we adaptively perturb the particles of the parameters in *ϕ*_*t*_ by choosing *σ*=2*%* of the value of the particle. We report the posterior estimates of all the state variables and model parameters using the method described in [50]. We detail this in Additional file 1.

## Additional file

Additional file 1 Supplementary Material for “SeqClone: Sequential Monte Carlo Based Inference of Tumor Subclones”. (PDF 203 kb)

## Abbreviations

CLL: Chronic lymphocytic leukemia
EM: Expectation maximization
IBP: Indian buffet process
MCMC: Markov chain Monte Carlo
PDF: Probability density function
PMF: Probability mass function
SMC: Sequential Monte Carlo
SNV: Single nucleotide variant
VAF: Variant allele fraction
WES: Whole exome sequencing
WGS: Whole genome sequencing
