# Supplementary material for: SeqClone: sequential Monte Carlo based inference of tumor subclones
Source: BMC Bioinformatics. 2019 Jan 5;20:6. doi: 10.1186/s12859-018-2562-y (PMC6320595; doi:10.1186/s12859-018-2562-y)
Supplement: Supplementary file 1 — Supplementary Material for “SeqClone: Sequential Monte Carlo Based Inference of Tumor Subclones”. (PDF 203 kb) [file 12859_2018_2562_MOESM1_ESM.pdf]

Supplementary Material for "SeqClone: Sequential Monte Carlo Based Inference of Tumor Subclones"

Oyetunji Ogundijo [1] and Xiaodong Wang [1,*]

Section A

Here, we present how the point estimates of the unknown variables in our model are obtained from the posterior weighted Monte Carlo samples from SeqClone, following the procedures highlighted in [1].

First, we factorize the joint posterior distribution of all the unknown variables given the input dataset (matrices $\mathbf{Y}$ and $\mathbf{V}$) as follows:

$$p(C, \mathbf{Z}, \mathbf{W}, p|\mathbf{Y}, \mathbf{V}) = p(C|\mathbf{Y}, \mathbf{V})p(\mathbf{Z}|\mathbf{Y}, \mathbf{V}, C)p(\mathbf{W}, p|\mathbf{Y}, \mathbf{V}C, \mathbf{Z}).$$

Then, based on the available posterior Monte Carlo samples for $\mathbf{Z}$, we approximately evaluate the marginal posterior distribution for C and then determine the maximum a posteriori (MAP) estimate $\hat{C}$.

Next, conditional on $\hat{C}$, we then estimate $\mathbf{Z}$ as follows. For any two matrices $\mathbf{Z}$ and $\mathbf{Z}'$, $1 \leq c, c' \leq \hat{C}$, define

$$\mathcal{D}_{cc'}(\mathbf{Z}, \mathbf{Z}') = \sum_{t=1}^{T} |z_{tc} - z'_{tc'}|,$$

and define a distance

$$d(\mathbf{Z}, \mathbf{Z}') = \min \sum_{c=1}^{\hat{C}} \mathcal{D}_{c,\pi_c}(\mathbf{Z}, \mathbf{Z}'),$$

where π_c is a permutation of $\{1, ..., \hat{C}\}$ and the minimum is over all possible permutations. Thus, an estimate for $\mathbf{Z}$ is defined as:

$$\hat{\mathbf{Z}} = \underset{\mathbf{Z}'}{\operatorname{argmin}} \int d(\mathbf{Z}, \mathbf{Z}') dp(\mathbf{Z}|\mathbf{Y}, \hat{C})$$
$$\approx \underset{\mathbf{Z}'}{\operatorname{argmin}} \sum_{i=1}^{N} w_T^i d(\mathbf{Z}_T^i, \mathbf{Z}')$$

*to whom correspondence should be addressed

Table 1: CLL077: Genotypes of Subclones.

		Clomial				BayClone				Cloe			
	Gene	C1	C2	C3	C4	C1	C2	C3	C4	C1	C2	C3	C4
1	BCL2L13	1	0	1	1	1	1	1	1	1	1	1	1
2	COL24A1	0	1	0	0	0	1	0	0	0	1	0	0
3	DAZAP1	0	0	1	1	0	0	1	1	0	0	1	1
4	EXOC6B	0	0	1	1	0	0	1	1	0	0	1	1
5	GHDC	0	0	1	1	0	0	1	1	0	0	1	1
6	GPR158	1	0	1	1	1	1	1	1	1	1	1	1
7	HMCN1	0	1	0	0	0	1	0	0	0	1	0	0
8	KLHDC2	0	1	0	0	0	1	0	0	0	1	0	0
9	LRRC16A	0	0	0	1	0	0	0	1	0	0	0	1
10	MAP2K1	0	1	0	0	0	1	0	0	0	1	0	0
11	NAMPTL	1	0	1	1	1	1	1	1	1	1	1	1
12	NOD1	0	1	0	0	0	1	0	0	0	1	0	0
13	OCA2	0	0	1	1	0	0	1	1	0	0	1	1
14	PLA2G16	0	0	1	1	0	0	1	1	0	0	1	1
15	SAMHD1	1	1	1	1	1	1	1	1	1	1	1	1
16	SLC12A1	1	0	1	1	1	1	1	1	1	1	1	1

for posterior Monte Carlo samples, $\{\mathbf{Z}_T^i\}_{i=1}^N$ and the normalized weights $\{w_i\}_{i=1}^N$.

Finally, we report posterior estimates $\hat{\mathbf{W}}$ and $\hat{p}$ for $\mathbf{W}$ and p, respectively conditional on $\hat{C}$ and $\hat{\mathbf{Z}}$.

Section B

In our experiments, for SeqClone, we set $N = 500$ for the CLL datasets, $N = 1000$ for the simulated datasets, $a_0 = 0.2$, $a = 3$, $a_{00} = 1$, and $b_{00} = 100$. α was set to 0.02 and 0.05 for the simulated and the CLL datasets, respectively. For Bayclone and Cloe, we ran $30,000$ iterations, discarding the first $15,000$ as burn-in, and thinning the chain by taking every 30th sample. For Clomial, we used 2000 iterations for all experiments.

Moreover, in Tables 1 - 6, we provide the results obtained on each of the CLL datasets when analyzed with Clomial, BayClone and Cloe, the three algorithms compared with SeqClone in the main paper. In the genotype matrices, each column denotes a subclone, and a 0 and a 1 denote the presence and absence of a mutation in a subclone, respectively. Also, the subclonal proportion matrices show the proportions of each subclone in each of the samples. For BayClone, we report a 1 in the genotype matrix for any output that is greater or equal to 0.5.

Table 2: CLL077: Proportions of Subclones.

		TP **a**	TP **b**	TP **c**	TP **d**	TP **e**
	C0	0.00	0.00	0.00	0.00	0.00
	C1	0.27	0.18	0.16	0.23	0.43
Clomial	C2	0.01	0.03	0.04	0.12	0.34
	C3	0.39	0.27	0.30	0.26	0.13
	C4	0.33	0.52	0.50	0.39	0.10
	C0	0.06	0.04	0.00	0.04	0.37
	C1	0.21	0.14	0.15	0.14	0.07
BayClone	C2	0.00	0.03	0.05	0.16	0.33
	C3	0.40	0.25	0.31	0.28	0.13
	C4	0.33	0.54	0.49	0.38	0.10
	C0	0.05	0.04	0.00	0.03	0.39
	C1	0.16	0.15	0.17	0.15	0.03
Cloe	C2	0.02	0.02	0.02	0.14	0.34
	C3	0.42	0.21	0.34	0.31	0.14
	C4	0.35	0.58	0.47	0.37	0.10

Table 3: CLL006: Genotypes of Subclones.

		Clomial				BayClone					Cloe					
	Gene	C1	C2	C3	C4	C1	C2	C3	C4	C5	C1	C2	C3	C4	C5	C6
1	ARHGAP29	1	1	1	1	1	1	1	1	1	1	1	1	1	1	1
2	EGFR	1	1	1	1	1	1	1	1	1	1	1	1	1	1	0
3	IRF4	0	0	0	1	1	0	0	0	1	1	0	0	0	1	0
4	KIAA0182	1	1	1	1	1	1	1	1	1	1	1	1	1	1	1
5	KIAA0319L	1	0	0	1	1	0	0	1	1	1	0	0	1	1	0
6	KLHL4	1	1	1	1	1	1	1	1	1	1	1	1	1	1	1
7	MED12	1	1	1	1	1	1	1	1	1	1	1	1	1	1	1
8	PILRB	1	1	1	1	1	1	1	1	1	1	1	1	1	1	1
9	RBPJ	1	0	0	0	1	0	0	0	0	1	0	0	0	0	0
10	SIK1	1	1	1	1	1	1	1	1	1	1	1	1	1	1	1
11	U2AF1	1	1	0	1	1	1	0	1	1	1	1	0	1	1	0

References

[1] Lee,J., et al. (2015) A Bayesian feature allocation model for tumor heterogeneity. *The Annals of Applied Statistics*, **9**, 621–639.

Table 4: CLL006: Proportions of Subclones.

		TP **a**	TP **b**	TP **c**	TP **d**	TP **e**
	C0	0.00	0.00	0.00	0.00	0.00
	C1	0.03	0.09	0.03	0.13	0.12
Clomial	C2	0.36	0.31	0.36	0.22	0.13
	C3	0.32	0.09	0.30	0.06	0.09
	C4	0.30	0.52	0.31	0.58	0.66
	C0	0.00	0.01	0.01	0.00	0.00
	C1	0.01	0.09	0.02	0.12	0.14
BayClone	C2	0.33	0.32	0.33	0.23	0.15
	C3	0.30	0.08	0.29	0.07	0.09
	C4	0.13	0.06	0.10	0.14	0.06
	C5	0.23	0.44	0.25	0.44	0.56
	C0	0.00	0.00	0.00	0.00	0.00
	C1	0.01	0.07	0.03	0.14	0.14
Cloe	C2	0.34	0.39	0.35	0.24	0.15
	C3	0.23	0.04	0.22	0.03	0.06
	C4	0.12	0.04	0.08	0.17	0.05
	C5	0.25	0.45	0.25	0.40	0.54
	C6	0.05	0.01	0.07	0.02	0.06

Table 5: CLL003: Genotypes of Subclones.

		Clomial				BayClone		Cloe			
	Gene	C1	C2	C3	C4	C1	C2	C1	C2	C3	C4
1	ADAD1	1	1	1	1	1	1	1	1	1	1
2	AMTN	0	1	0	1	0	1	0	1	0	0
3	APBB2	0	1	0	1	0	1	0	1	0	0
4	ASXL1	1	0	0	1	1	0	1	0	0	1
5	ATM	0	1	1	1	0	1	0	1	0	0
6	BPIL2	0	1	0	1	0	1	0	1	0	0
7	CHRNB2	1	0	0	0	1	0	1	0	0	0
8	CHTF8	1	1	1	1	1	1	1	1	1	1
9	FAT3	1	0	0	0	1	0	1	0	0	0
10	HERC2	1	1	1	1	1	1	1	1	1	1
11	IL11RA	1	1	0	1	1	1	1	1	1	0
12	MTUS1	0	1	0	1	0	1	0	1	0	0
13	MUSK	1	0	0	1	1	0	1	0	0	1
14	NPY	1	0	0	0	1	0	1	0	0	0
15	NRG3	1	0	0	0	1	0	1	0	0	0
16	PLEKHG5	0	1	0	1	0	1	0	1	0	0
17	SEMA3E	1	0	0	1	1	0	1	0	0	1
18	SF3B1	1	1	1	1	1	1	1	1	1	1
19	SHROOM1	1	1	1	1	1	1	1	1	1	1
20	SPTAN1	0	1	0	1	0	1	0	1	0	0

Table 6: CLL003: Proportions of Subclones.

		TP **a**	TP **b**	TP **c**	TP **d**	TP **e**
	C0	0.00	0.00	0.35	0.09	0.03
	C1	0.00	0.01	0.46	0.91	0.96
Clomial	C2	0.72	0.84	0.01	0.00	0.00
	C3	0.18	0.08	0.06	0.00	0.00
	C4	0.10	0.07	0.12	0.01	0.01
	C0	0.00	0.01	0.33	0.01	0.02
	C1	0.06	0.03	0.45	0.96	0.96
BayClone	C2	0.94	0.96	0.13	0.03	0.02
	C0	0.08	0.00	0.34	0.06	0.01
	C1	0.01	0.00	0.45	0.94	0.98
Cloe	C2	0.77	0.85	0.10	0.00	0.00
	C3	0.00	0.09	0.00	0.00	0.01
	C4	0.14	0.06	0.11	0.00	0.00
